# Outcomes with and without postmastectomy radiotherapy for pT3N0‐1M0 breast cancer: An institutional experience

**DOI:** 10.1002/cam4.6927

**Published:** 2024-01-08

**Authors:** Xinxin Rao, Xuanyi Wang, Kairui Jin, Yilan Yang, Xu Zhao, Zhe Pan, Weiluo Lv, Zhen Zhang, Li Zhang, Xiaoli Yu, Xiaomao Guo

**Affiliations:** ^1^ Department of Radiation Oncology Fudan University Shanghai Cancer Center Shanghai China; ^2^ Department of Oncology, Shanghai Medical College Fudan University Shanghai China; ^3^ Shanghai Key Laboratory of Radiation Oncology Shanghai China

**Keywords:** breast cancer, postmastectomy radiotherapy, prognostic factors, retrospective analysis

## Abstract

**Aim:**

The objective of this study is to comprehensively evaluate the therapeutic efficacy of postmastectomy radiotherapy (PMRT) in treating patients with pT3N0‐1M0 breast cancer within the context of modern therapeutic strategies.

**Methods:**

Clinical data from patients with pT3N0‐1M0 breast cancer who underwent mastectomy from January 2005 to December 2018 at our institution were retrospectively analyzed.

**Results:**

The study involved a total of 222 participants, with 112 individuals undergoing PMRT and 110 individuals not receiving it. The median follow‐up duration was 77 months (range: 6–171 months). The entire cohort demonstrated 5‐year disease‐free survival (DFS) and overall survival (OS) rates of 85.1% and 91.0%, respectively, along with a locoregional recurrence (LRR) rate as low as 7.2%. The PMRT group showed significantly better 5‐year DFS (90.2% vs. 80.0%, *p* = 0.02) and OS (95.5% vs. 86.4%, *p* = 0.012) rates, as well as a lower LRR rate (4.5% vs. 10.0%, *p* = 0.122), compared to the group without PMRT. Cox regression analysis confirmed the independent prognostic significance of PMRT for both DFS (*p* = 0.040) and OS (*p* = 0.047). Following propensity score matching (PSM), the analysis included 100 matched patients, revealing an improved prognosis for those who received PMRT (DFS: *p* = 0.067; OS: *p* = 0.043).

**Conclusions:**

Our study reveals favorable prognoses for pT3N0‐1M0 breast cancer patients treated within contemporary therapeutic approaches. The pivotal role of PMRT in this context is evident. However, due to the retrospective design of our study and the relatively limited sample size, further investigation is imperative to validate and enhance these initial findings.

## Graphical abstract

Postmastectomy radiotherapy (PMRT) improves survival in patients with pT3N0‐1M0 breast cancer. PMRT is an independent prognostic factor for DFS and OS in pT3N0‐1M0 breast cancer. The impact of PMRT appears to vary among patients with different risk statuses.

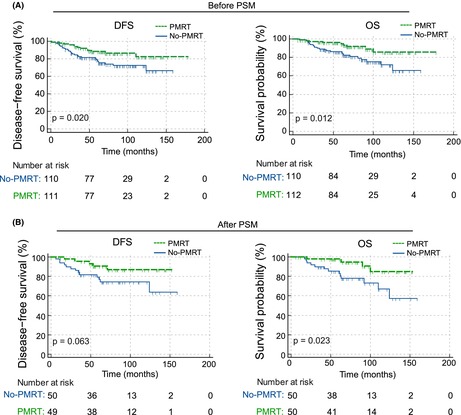

## 1 INTRODUCTION

Approximately two decades ago, well‐conducted randomized controlled trials, including DBCG 82b,^1^ DBCG 82c,^2^ and the British Columbia Trial,^3^, ^4^ provided compelling evidence supporting improved survival outcomes associated with postmastectomy radiotherapy (PMRT) in high‐risk breast cancer patients. However, the role of PMRT in primary breast malignancies (tumor size >5 cm) with 0–3 positive lymph nodes (pT3N0‐1M0) remains controversial due to conflicting results from previous retrospective studies, especially for pT3N0M0 breast cancer.^5^, ^6^, ^7^, ^8^, ^9^ The rarity of pT3N0‐1M0 breast cancer patients,^1^, ^2^, ^10^ attributed to the positive association between tumor size and the risk of lymph node disease,^11^, ^12^ has limited the availability of large‐scale prospective trials focused on this specific patient subset.

In the modern era, the dramatic development of systemic therapy for breast cancer has demonstrated superior efficacy in managing both local and distant disease.^13^, ^14^ Consequently, present‐day patients are expected to have a lower absolute risk of recurrence compared to their counterparts in the past. However, the actual advantage of PMRT for individuals diagnosed with pT3N0‐1M0 breast cancer is still unclear when considering contemporary treatment protocols.

In this study, we sought to address the existing research gap by retrospectively analyzing the effects of PMRT in patients diagnosed with pT3N0‐1M0 breast cancer from January 2005 to December 2018, a period characterized by the application of contemporary systemic treatment modalities. Comparative analyses were performed to evaluate the outcomes between patients with and without PMRT.

## 2 METHODS

### 2.1 Patients

This retrospective study utilized tumor registries from the Fudan University Shanghai Cancer Center and obtained ethical approval from our institute's Ethics Committee. The registries encompassed the period between January 2005 and December 2018, identifying a total of 35,774 breast cancer patients treated at our institution. Inclusion and exclusion criteria are provided in Figure S1. Our analysis focused exclusively on female patients who had undergone mastectomy and met the following criteria: a primary tumor size exceeding 5 cm, absence of lymph node involvement, or presence of 1–3 positive nodes. We excluded patients with unclear or unspecified surgical resections, unknown pT‐stages, absent regional nodal evaluations, metastatic disease at the time of diagnosis, uncertain chemotherapy/radiotherapy status, histories of previous malignancies, bilateral second/synchronous bilateral breast cancer, less than 6 months of follow‐up, and neoadjuvant therapy. Eventually, we retrospectively assessed a cohort of 222 female patients with pT3N0‐1M0 primary breast cancer. These patients were categorized based on whether they received PMRT. Pathological data from all patients were verified in the central laboratory of the pathology department at our institution.

### 2.2 Follow‐up and outcomes

Regular follow‐up assessments were conducted for study participants, which included physical examination, laboratory tests, and various imaging inspections, such as CT, ultrasound, and MRI, aimed at detecting disease recurrence. The primary endpoint of this study included overall survival (OS), locoregional recurrence‐free survival, and distant metastasis‐free survival. The definition of OS was the duration in months from pathological diagnosis to death or the last follow‐up. Locoregional recurrence (LRR) was identified as cancer reappearance or progression in the chest wall or the same‐side breast, as well as regional nodal stations including the internal mammary nodals (IMNs), ipsilateral axillary lymph nodes, or supraclavicular lymph nodes. Recurrences at any other sites were categorized as distant metastases (DM). Disease‐free survival (DFS) was calculated from pathological diagnosis date to the occurrence of death, LRR, DM, or the last recorded visit.

### 2.3 Propensity score matching (PSM)

Using the MatchIt package in R (version 4.1.1), PSM aimed to address imbalances in vital baseline characteristics between the two groups. Covariates matched in the PSM analysis included age, pN stage, diagnosis year, histologic grade, tumor size, lymph vascular invasion (LVI) status, molecule subtype, as well as the receipt of chemotherapy, anti‐HER2 therapy, and hormone therapy. The “nearest neighbor” method with a 1:1 ratio and a caliper of 0.2 was utilized to optimize the matching process and achieve a balanced comparison between the two groups.

### 2.4 Statistical analysis

Categorical clinical characteristics were compared using Chi‐squared or Fisher's tests, as appropriate. Survival disparities were assessed using the Kaplan–Meier method, and log‐rank tests were conducted. Graphical illustrations were generated using MedCalc 19.5.6. Prognostic factors were analyzed through Cox regression analyses. Univariate analysis excluded cases with unspecified pathological information. The multivariate analysis considered variables with univariate *p*‐values <0.1, along with clinically relevant variables (pN stage, ER, PR, and HER2 status, chemotherapy). For data analysis, we utilized SPSS 26.0 and R 4.1.1 as the software platforms. Statistical significance was determined at a two‐tailed *p*‐value of <0.05.

## 3 RESULTS

### 3.1 Patient characteristics and treatments

Overall, 222 patients participated in this retrospective analysis, of which 112 were treated with PMRT while the remaining 110 were not. Table 1 presents a comprehensive display of the clinical and pathological characteristics in accordance with their medical records. Baseline characteristics, including pN stage, diagnosis year, histologic grade, tumor location, laterality, tumor size, ER/PR status, and human epidermal growth factor receptor 2 (HER‐2) expression, were well‐balanced between the two groups, as presented in Table 1 (left column). The median age of the entire patient cohort was 49 years (range: 27–83 years). Notably, patients who underwent PMRT (median 45 years; range: 27–80 years) were significantly younger compared to the no‐PMRT group (median 53 years; range: 28–83 years) (*p* < 0.001). The PMRT group also showed a significantly higher utilization of systemic treatments, including endocrine therapy, anti‐HER2 targeted therapy, and adjuvant chemotherapy (*p* < 0.001, *p* = 0.007, *p* < 0.001, respectively), compared to the no‐PMRT group. To address potential imbalances arising from clinicians' preferences, PSM was applied. After PSM, 100 patients were successfully matched in a 1:1 ratio, achieving balanced clinical baseline characteristics (Table 1, right column).

**TABLE 1:** Demographic and clinical characteristics.

Characteristics	Before PSM				After PSM			
	Total (*n* = 222)	PMRT (*n* = 112)	No‐PMRT (*n* = 110)	*p*‐value	Total (*n* = 100)	PMRT (*n* = 50)	No‐PMRT (*n* = 50)	*p*‐value
	Number (%)	Number (%)	Number (%)		Number (%)	Number (%)	Number (%)	
Age				<0.001***				0.295
Median (range)	49 (27–83)	45 (27–80)	53 (28–83)		49.5 (27–80)	47 (27–80)	50 (28–76)	
≤40	52 (23.4%)	39 (34.8%)	13 (11.8%)		23 (23.0%)	13 (26.0%)	10 (20.0%)	
41–60	125 (56.3%)	60 (53.6%)	65 (59.1%)		60 (60.0%)	29 (58.0%)	31 (62.0%)	
>60	45 (20.3%)	13 (11.6%)	32 (29.1%)		17 (17.0%)	8 (16.0%)	9 (18.0%)	
pN stage				0.676				0.689
N0	122 (55.0%)	60 (53.6%)	62 (56.4%)		48 (48.0%)	25 (50.0%)	23 (46.0%)	
N1	100 (45.0%)	52 (46.4%)	48 (43.6%)		52 (52.0%)	25 (50.0%)	27 (54.0%)	
Diagnosis year				0.267				0.967
2005–2010	50 (22.5%)	23 (20.5%)	27 (24.5%)		29 (29.0%)	15 (30.0%)	14 (28.0%)	
2011–2014	67 (30.2%)	30 (26.8%)	37 (33.6%)		31 (31.0%)	15 (30.0%)	16 (32.0%)	
2015–2018	105 (47.3%)	59 (52.7%)	46 (41.8%)		40 (40.0%)	20 (40.0%)	20 (40.0%)	
Histologic grade				0.704				0.920
I–II	115 (51.8%)	55 (49.1%)	60 (54.5%)		56 (56.0%)	29 (58.0%)	27 (54.0%)	
III	100 (45.0%)	53 (47.3%)	47 (42.7%)		42 (42.0%)	20 (40.0%)	22 (44.0%)	
Unknown	7 (3.2%)	4 (3.6%)	3 (3.6%)		2 (2.0%)	1 (2.0%)	1 (2.0%)	
Tumor location				0.577				0.224
Lateral	125 (56.3%)	61 (54.5%)	64 (58.2%)		58 (58.0%)	26 (52.0%)	32 (64.0%)	
Central/medial	97 (43.7%)	51 (45.5%)	46 (41.8%)		42 (42.0%)	24 (48.0%)	18 (36.0%)	
Laterality				0.691				0.689
Left	106 (47.7%)	52 (46.4%)	54 (49.1%)		50 (50.0%)	24 (48.0%)	26 (52.0%)	
Right	116 (52.3%)	60 (53.6%)	56 (50.9%)		50 (50.0%)	26 (52.0%)	24 (48.0%)	
Tumor size				0.164				0.483
Median (range)	6.0 (5.1–30.0)	6.0 (5.1–25.0)	6.0 (5.2–30.0)		6.4 (5.1,20.0)	6.5 (5.1–20.0)	6.0 (5.2–18.0)	
5.1–7.0 cm	163 (73.4%)	89 (79.5%)	74 (67.3%)		73 (73.0%)	36 (72.0%)	37 (74.0%)	
>7.0 cm	59 (26.6%)	23 (20.5%)	36 (32.7%)		27 (27.0%)	14 (28.0%)	13 (26.0%)	
LVI				0.104				0.689
Positive	103 (46.4%)	58 (51.8%)	45 (40.9%)		50 (50.0%)	26 (52.0%)	24 (48.0%)	
Negative	119 (53.6%)	54 (48.2%)	65 (59.1%)		50 (50.0%)	24 (48.0%)	26 (52.0%)	
Margin				0.656				0.497
Positive	3 (1.4%)	1 (0.9%)	2 (1.8%)		1 (2.0%)	1 (2.0%)	0 (0.0%)	
Negative	209 (94.1%)	107 (95.5%)	102 (92.7%)		93 (93.0%)	46 (92.0%)	47 (94.0%)	
Unknown	10 (4.5%)	4 (3.6%)	6 (5.5%)		6 (6.0%)	3 (6.0%)	3 (6.0%)	
ER status				0.259				0.839
Positive	137 (61.7%)	73 (65.2%)	64 (58.2%)		59 (59.0%)	29 (58.0%)	30 (60.0%)	
Negative	84 (37.8%)	39 (34.8%)	45 (40.9%)		41 (41.0%)	21 (42.0%)	20 (40.0%)	
Unknown	1 (0.5%)	0 (0%)	1 (0.9%)		0 (0.0%)	0 (0.0%)	0 (0.0%)	
PR status				0.447				0.689
Positive	111 (50%)	56 (50.0%)	55 (50.0%)		52 (52.0%)	25 (50.0%)	27 (54.0%)	
Negative	110 (49.5%)	56 (50.0%)	54 (49.1%)		48 (48.0%)	25 (50.0%)	23 (46.0%)	
Unknown	1 (0.5%)	0 (0%)	1 (0.9%)		0 (0.0%)	0 (0.0%)	0 (0.0%)	
HER2 status				0.439				0.828
Positive	85 (38.3%)	46 (41.1%)	39 (35.5%)		43 (43.0%)	23 (46.0%)	20 (40.0%)	
Negative	114 (51.4%)	57 (50.9%)	57 (51.8%)		44 (44.0%)	21 (42.0%)	23 (46.0%)	
Unknown	23 (10.4%)	9 (8.0%)	14 (16.4%)		13 (13.0%)	6 (12.0%)	7 (14.0%)	
Molecular subtype				0.122				0.959
Luminal	141 (63.5%)	77 (68.8%)	64 (58.2%)		61 (61.0%)	31 (62.0%)	30 (60.0%)	
HER2+	43 (19.4%)	18 (16.1%)	25 (22.7%)		24 (24.0%)	12 (24.0%)	12 (24.0%)	
TNBC	36 (16.2%)	17 (15.2%)	19 (17.3%)		15 (15.0%)	7 (14.0%)	8 (16.0%)	
Unknown	2 (0.9%)	0 (0%)	2 (1.8%)		0 (0.0%)	0 (0.0%)	0 (0.0%)	
Chemotherapy				<0.001***				1.000
Yes	153 (68.9%)	99 (88.4%)	54 (49.1%)		84 (84.0%)	42 (84.0%)	42 (84.0%)	
No	69 (31.1%)	13 (11.6%)	56 (50.9%)		16 (16.0%)	8 (16.0%)	8 (16.0%)	
Anti‐HER2 therapy				0.007*				0.476
Yes	35 (15.8%)	25 (22.3%)	10 (9.1%)		23 (23.0%)	13 (26.0%)	10 (20.0%)	
No	187 (84.2%)	87 (77.7%)	100 (90.9%)		77 (77.0%)	37 (74.0%)	40 (80.0%)	
Hormone therapy				<0.001***				0.317
Yes	108 (48.6%)	71 (63.4%)	37 (33.6%)		49 (49.0%)	27 (54.0%)	22 (44.0%)	
No	114 (51.4%)	41 (36.6%)	73 (66.4%)		51 (51.0%)	23 (46.0%)	28 (56.0%)	

Abbreviations: ER, estrogen receptor; HER2, human epidermal growth factor receptor 2; LVI, lymph vascular invasion; PMRT, postmastectomy radiotherapy; pN stage, pathologic lymph node stage; PR, progesterone receptor; PSM, propensity score matching; TNBC, triple‐negative breast cancer.

*** *p* < 0.001.

* *p* < 0.05.

All enrolled participants (*n* = 222) in this study underwent mastectomy, with the majority undergoing axillary lymph node dissection (*n* = 159, 71.6%), while a smaller subset underwent sentinel lymph node biopsy only (*n* = 63, 28.4%). Adjuvant chemotherapy was administered to 68.9% (153 patients) of the total cohort. Among these patients, the majority (52.9%, 81/153) received a combination of anthracycline and taxane chemotherapy, 24.8% (38/153) of patients only received anthracycline‐based chemotherapy, while 16.3% (25/153) of patients received taxane‐based chemotherapy exclusively. Hormonal therapy including tamoxifen or aromatase inhibitor was delivered to 76.6% (108/141) of patients who tested positive for estrogen receptor (ER) and/or progesterone receptor (PR). For HER‐2–positive patients, trastuzumab was recommended for all, and 41.2% (35/85) actually received trastuzumab as part of their treatment regimen.

In the PMRT subset, a significant proportion (82, 73.2%) of individuals were administered intensity‐modulated radiation therapy (IMRT). A standardized dose of 50 Gy was uniformly administered in 25 fractions across all study participants. The chest wall was irradiated for all patients, while the irradiation of the supraclavicular or infraclavicular lymph nodal region and the IMN region was at the discretion of physicians. Generally, irradiation to the regional lymph node areas was delivered to patients with indicators of potential regional recurrence, such as lymph node involvement or LVI. Specifically, chest wall irradiation alone was delivered to 35 (31.3%) patients, and 77 (68.7%) patients received combined chest wall and regional lymph node irradiation.

### 3.2 Survival analysis

**FIGURE 1:**
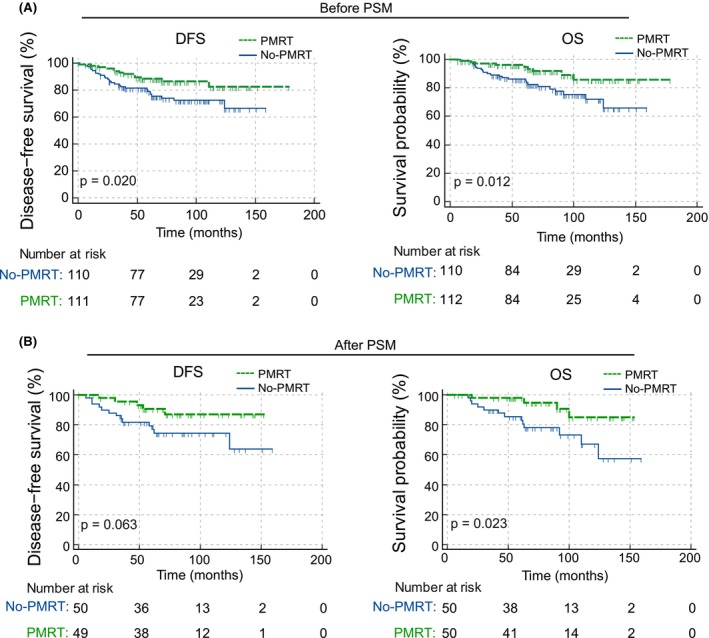
Kaplan–Meier curves illustrating disease‐free survival (DFS) and overall survival (OS) for (A) the entire patient cohort and (B) propensity score‐matched patient subsets, categorized by postmastectomy radiotherapy utilization. Patients at risk were also shown.

With a median follow‐up of 77 months (range, 6–171 months), tumor recurrence occurred in 31 patients, and 33 patients died. The entire cohort exhibited estimated 5‐year DFS and OS rates of 85.1% and 91.0%, respectively. Notably, the PMRT group showed a higher 5‐year DFS rate (90.2%) compared to the no‐PMRT group (80.0%) (*p* = 0.02; Figure 1, above). Correspondingly, the 5‐year OS rates were 95.5% for the PMRT group and 86.4% for the no‐PMRT group (*p* = 0.012; Figure 1, above). Cox multivariate analysis demonstrated PMRT's independent prognostic significance for both DFS and OS (Table 2; DFS: HR = 0.47, 95% CI 0.22–0.97, *p* = 0.040; OS: HR = 0.41, 95% CI 0.17–0.99, *p* = 0.047). Additionally, the presence of LVI independently predicted poorer DFS outcomes (*p* = 0.031).

**TABLE 2:** Univariate and multivariate analysis for DFS and OS before PSM.

Variables	DFS						OS					
	Univariate analysis			Multivariate analysis			Univariate analysis			Multivariate analysis		
	HR	95% CI	*p*‐value	HR	95% CI	*p*‐value	HR	95% CI1	*p*‐value	HR	95% CI	*p*‐value
PMRT												
Yes vs. no	0.47	0.24–0.90	0.023*	0.47	0.22–0.97	0.040*	0.39	0.18–0.83	0.015*	0.41	0.17–0.99	0.047*
Age												
41–60 vs. ≤40	0.68	0.31–1.48	0.331	‐	‐	‐	1.47	0.49–4.45	0.492	1.08	0.34–3.42	0.900
>60 vs. ≤40	1.74	0.77–3.93	0.181	‐	‐	‐	4.52	1.49–13.8	0.008*	2.97	0.85–10.3	0.087
pN stage												
N0 vs. N1	0.52	0.28–0.97 0.82	0.039*	0.77	0.35–1.69	0.511	0.50	0.25–1.00	0.051	0.74	0.32–1.75	0.495
Diagnosis year												
2011–2014 vs. 2005–2010	1.02	0.45–2.28	0.971	‐	‐	‐	1.01	0.42–2.38	0.992	‐	‐	‐
2015–2018 vs. 2005–2010	1.08	0.49–2.35	0.857	‐	‐	‐	1.15	0.49–2.83	0.759	‐	‐	‐
Tumor size												
5–7 cm vs. >7 cm	1.05	0.52–2.15	0.887	‐	‐	‐	0.72	0.34–1.52	0.388	‐	‐	‐
Histologic grade												
I–II vs. II–III	0.78	0.42–1.44 1.32	0.423	‐	‐	‐	0.75	0.38–1.49	0.415	‐	‐	‐
Laterality												
Left vs. right	0.86	0.46–1.59	0.630	‐	‐	‐	0.71	0.35–1.42	0.330	‐	‐	‐
Location												
Lateral vs. central/medial	0.69	0.37–1.27	0.232	‐	‐	‐	0.52	0.26–1.05	0.067	0.57	0.28–1.15	0.115
ER												
Positive vs. negative	0.61	0.33–1.13	0.114	0.48	0.17–1.36	0.167	0.60	0.30–1.18	0.138	0.32	0.09–1.22	0.095
PR												
Positive vs. negative	0.75	0.40–1.39	0.360	1.18	0.41–3.34	0.762	0.87	0.44–1.72	0.689	2.14	0.57–8.12	0.263
HER2												
Positive vs. negative	0.86	0.44–1.69	0.664	0.75	0.37–1.53	0.427	0.57	0.26–1.24	0.155	0.63	0.27–1.47	0.288
LVI												
Positive vs. negative	2.36	1.25–4.46	0.008*	2.47	1.09–5.63	0.031*	2.18	1.08–4.39	0.030*	2.30	0.95–5.53	0.064
Chemotherapy												
Yes vs. no	0.76	0.40–1.46	0.409	0.91	0.44–1.89	0.794	0.84	0.40–1.77	0.640	1.35	0.59–3.11	0.481

Abbreviations: 95% CI, 95% confidence interval; DFS, disease‐free survival; ER, estrogen receptor; HER2, human epidermal growth factor receptor 2; HR, hazard ratio; LVI, lymph vascular invasion; OS, overall survival; PMRT, postmastectomy radiotherapy; pN stage, pathologic lymph node stage; PR, progesterone receptor; PSM, propensity score matching.

* *p* < 0.05.

After PSM, PMRT still demonstrated significant improvements in 5‐year OS (97.8% vs. 85.5%, *p* = 0.023) and showed a borderline improvement in DFS (90.5% vs. 79.4%, *p* = 0.063; Figure 1, below). Adjusted hazard ratios for PMRT were 0.38 (95% CI 0.13–1.07, *p* = 0.067) for DFS and 0.29 (95% CI 0.09–0.96, *p* = 0.043) for OS, reinforcing the prognostic significance of PMRT (Table 3). Moreover, advanced age (>60 years) was correlated with decreased OS (*p* = 0.034).

**TABLE 3:** Univariate and multivariate analysis for DFS and OS after PSM.

Variables	DFS						OS					
	Univariate analysis			Multivariate analysis			Univariate analysis			Multivariate analysis		
	HR	95% CI	*p*‐value	HR	95% CI	*p*‐value	HR	95% CI1	*p*‐value	HR	95% CI	*p*‐value
PMRT												
Yes vs. no	0.39	0.14–1.09	0.074	0.38	0.13–1.07	0.067	0.29	0.10–0.90	0.032*	0.29	0.09–0.96	0.043*
Age												
41–60 vs. ≤40	0.58	0.17–1.99	0.383	‐	‐	‐	1.01	0.21–4.92	0.991	0.91	0.18–4.64	0.909
>60 vs. ≤40	2.58	0.75–8.86	0.132	‐	‐	‐	7.18	1.50–34.4	0.014*	5.68	1.14–28.3	0.034*
pN stage												
N0 vs. N1	0.63	0.24–1.64	0.341	0.65	0.20–2.15	0.479	0.68	0.26–1.80	0.434	0.74	0.22–2.50	0.631
Diagnosis year												
2011–2014 vs. 2005–2010	1.20	0.40–3.59	0.742	‐	‐	‐	0.87	0.28–2.78	0.820	‐	‐	‐
2015–2018 vs. 2005–2010	0.84	0.25–2.82	0.776	‐	‐	‐	1.28	0.36–4.53	0.699	‐	‐	‐
Tumor size												
5–7 cm vs. >7 cm	0.85	0.30–2.38	0.754	‐	‐	‐	0.64	0.22–1.85	0.412	‐	‐	‐
Histologic grade												
I–II vs. II–III	1.03	0.40–2.65 1.32	0.959	‐	‐	‐	1.15	0.43–3.13	0.779	‐	‐	‐
Laterality												
Left vs. right	1.03	0.41–2.59	0.952	‐	‐	‐	0.96	0.37–2.50	0.938	‐	‐	‐
Location												
Lateral vs. central/medial	0.57	0.22–1.44	0.233	‐	‐	‐	0.51	0.19–1.34	1.171	‐	‐	‐
ER												
Positive vs. negative	0.84	0.33–2.14	0.717	0.72	0.11–4.83	0.732	0.86	0.33–2.24	0.756	0.30	0.02–4.24	0.373
PR												
Positive vs. negative	0.89	0.35–2.24	0.803	0.90	0.13–6.19	0.914	1.10	0.43–2.87	0.839	2.14	0.15–31.4	0.578
HER2												
Positive vs. negative	0.65	0.23–1.83	0.411	0.61	0.20–1.89	0.395	1.02	0.20–5.28	0.983	0.59	0.17–2.06	0.409
LVI												
Positive vs. negative	1.38	0.54–3.54	0.500	1.17	0.35–3.90	0.799	1.18	0.45–3.12	0.734	0.96	0.28–3.28	0.948
Chemotherapy												
Yes vs. no	1.22	0.28–5.32	0.793	1.29	0.27–6.20	0.751	0.83	0.19–3.70	0.806	0.50	0.09–2.74	0.424

Abbreviations: 95% CI, 95% confidence interval; DFS, disease‐free survival; ER, estrogen receptor; HER2, human epidermal growth factor receptor 2; HR, hazard ratio; LVI, lymph vascular invasion; OS, overall survival; PMRT, postmastectomy radiotherapy; pN stage, pathologic lymph node stage; PR, progesterone receptor; PSM, propensity score matching.

* *p* < 0.05.

### 3.3 Subgroup analysis

**FIGURE 2:**
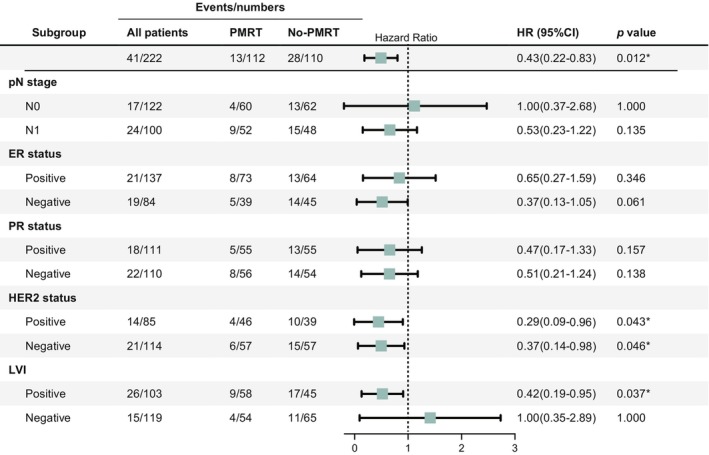
Adjusted hazard ratio (HR) of postmastectomy radiotherapy (PMRT) in different patient subgroups. Cox multivariate regression analysis for disease‐free survival (DFS) was performed. *p < 0.05.

To avoid overstating the impact of PMRT within a heterogeneous population, we further explored potential variations in its effects across distinct clinically relevant populations. Patients were stratified based on pN stage, molecular classification, and LVI status. Subsequent multivariable Cox regression analyses were performed within each subgroup to assess the prognostic value of PMRT (Figure 2). When stratifying by pN stage, PMRT did not demonstrate a significant prognostic impact in either the pT3N0M0 and pT3N1M0 subgroups. Upon stratification by ER status, a borderline improvement in DFS was noted with PMRT in the ER‐negative subgroup (HR, 0.37; 95% CI 0.13–1.05; *p* = 0.061), while no significant effect was observed in the ER‐positive subgroup (HR, 0.65; 95% CI 0.27–1.59; *p* = 0.346). A significantly enhanced DFS was identified with PMRT in both the HER2‐positive (HR, 0.29; 95% CI 0.09–0.96; *p* = 0.043) and HER2‐negative (HR, 0.37; 95% CI 0.14–0.98; *p* = 0.046) subgroups. However, PMRT did not yield a significant prognostic impact in either the PR‐positive or PR‐negative subgroups. After stratification according to LVI status, PMRT exhibited a significant prognostic impact in LVI‐positive subgroups (HR, 0.42; 95% CI 0.19–0.98; *p* = 0.037), but the prognostic effect of PMRT on LVI‐negative patients remains unclear (HR, 1.00; 95% CI 0.35–2.89; *p* = 1.000).

### 3.4 Patterns of recurrence

The recurrence pattern among pT3N0‐1M0 patients in the study was investigated and the incidences of relapse were documented (Table 4). Among the entire cohort, 31 patients (14%) experienced tumor recurrence, with 18 patients (16.4%) in the no‐PMRT group and 13 patients (11.6%) in the PMRT group. Distant metastasis emerged as the predominant type of recurrence, observed in 23 patients (10.4%), including 14 patients (12.7%) from the no‐PMRT group and nine patients (8.0%) from the PMRT group. Notably, PMRT reduced LRR by more than twofold (PMRT: 4.5% vs. no‐PMRT: 10.0%, *p* = 0.122). Specifically, the PMRT group demonstrated lower relapse rates in the ipsilateral breast or chest wall (PMRT: 2.7% vs. no‐PMRT: 7.3%) and a reduction in supraclavicular lymph node recurrence (PMRT: 0.9% vs. no‐PMRT: 2.7%). Recurrence in the IMNs was infrequent, with only one case observed in the no‐PMRT group (0.9%).

**TABLE 4:** Patterns of recurrence.

	Total (*n* = 222)	PMRT (*n* = 112)	No‐PMRT (*n* = 110)
	Number (%)	Number (%)	Number (%)
Any recurrence	31 (14.0%)	13 (11.6%)	18 (16.4%)
Distant metastasis	23 (10.4%)	9 (8.0%)	14 (12.7%)
Locoregional recurrence	16 (7.2%)	5 (4.5%)	11 (10.0%)
Local^a^	11 (5.0%)	3 (2.7%)	8 (7.3%)
Regional	9 (4.1%)	3 (2.7%)	6 (5.5%)
Supraclavicular lymph node	4 (1.8%)	1 (0.9%)	3 (2.7%)
Axillary lymph node	7 (3.2%)	3 (2.7%)	4 (3.6%)
IMN	1 (0.5%)	0 (0.0%)	1 (0.9%)

^a^ Local is defined here as relapsed disease within the ipsilateral breast or chest wall.

Abbreviations: IMN, internal mammary nodes; PMRT, postmastectomy radiotherapy.

## 4 DISCUSSION

In this study, we assessed DFS, OS and the recurrence pattern among patients diagnosed with pT3N0‐1M0 breast cancer in a real‐world setting, aiming to uncover the true therapeutic impact of PMRT within the context of modern systemic treatments. We also explored subgroups within this population that might potentially derive greater benefits from PMRT.

A meta‐analysis of 22 randomized trials, along with a subgroup analysis of the DBCG 82 b & c randomized trials, underscores the significant advantages of PMRT in reducing recurrence rates and breast cancer‐related mortality for patients with 1–3 positive lymph nodes. However, the survival benefit for patients with node‐negative disease appears less prominent.^15^, ^16^ Nevertheless, it is important to acknowledge that only a limited number of the participants enrolled in these clinical trials belonged to the pT3N0‐1M0 subgroup, introducing uncertainty regarding the potential benefits of PMRT for this specific subgroup. Additionally, the radiotherapy techniques and systemic therapies utilized in these studies are considered to be obsolete.

Due to the relatively low prevalence of pT3N0‐1M0 breast cancer, investigations focusing on this specific subgroup are primarily derived from retrospective studies or analyses utilizing population‐based databases. Floyd et al.^5^ reported favorable 5‐year LRR (7.6%) and DFS (86%) among pT3N0M0 breast cancer patients, highlighting the relevance of LVI as a poor prognostic factor for DFS and OS. In a comprehensive review, Taghian et al.^17^ documented a 7.2% LRR rate in pT3N0M0 breast cancer patients, without identifying statistically significant risk factors. Our study observed comparable rates, with 5‐year LRR at 7.2% and DFS at 85.1% in pT3N0‐1M0 breast cancer patients, and additionally identified LVI as an independent DFS predictor. Despite the limited occurrence of events, our study exhibited a substantial survival improvement with PMRT in pT3N0‐1M0 breast cancer patients, which is different from the two aforementioned studies. This discrepancy may be attributed to variations in patient populations and differences in treatment. The treatment period of the two previous studies were between 1981 and 2002, while our study examined patients treated between 2005 and 2018, during which more advanced systemic chemotherapy and radiotherapy techniques were utilized. Additionally, our study also included patients with pT3N1M0 breast cancer, expanding the scope of investigation.

The efficacy of PMRT in pT3N0‐1M0 breast cancer patients within the modern era has been demonstrated by several retrospective studies. Frandsen et al. exhibited a notable enhancement in the 5‐year OS rate among pT3N0M0 breast cancer patients who received PMRT compared to non‐recipients (83.7% vs. 79.8%, *p* < 0.001), utilizing the National Cancer Database.^18^ Another retrospective analysis indicated a significant OS improvement with PMRT among pT3N0M0 patients without adjuvant chemotherapy (74% vs. 65%, *p* < 0.001).^19^ In a recent multicenter retrospective study conducted in Korea (KROG 20–03, 2021),^20^ 274 pT3N0M0 breast cancer patients diagnosed between 2000 and 2016 across 18 institutions were examined. Notably, PMRT recipients were generally younger, had a higher proportion of PR‐positive tumors, and demonstrated increased utilization of adjuvant chemotherapy (*p* < 0.001, *p* = 0.018, and *p* < 0.001, respectively). The results revealed a substantial improvement in 8‐year DFS with PMRT (91.8% vs. 73.9%, *p* = 0.008). Multivariate analysis further indicated that the absence of LVI and PMRT held significant prognostic significance for improved DFS (*p* = 0.025 and *p* = 0.009, respectively).

These findings closely parallel to the results of our study, wherein the PMRT group exhibited a younger age distribution and a higher proportion of patients receiving endocrine therapy, adjuvant chemotherapy, and anti‐HER‐2 targeted therapy compared to the no‐PMRT group. The observed heightened 5‐year DFS and OS rates within the PMRT group further support the treatment value of PMRT in modern systemic treatment era. Our multivariable analysis outcomes also confirmed the independent prognostic significance of both PMRT and LVI for DFS. Worth noting is that, despite 45% of our cohort having pT3N1M0 breast cancer, our study achieved comparable or even improved DFS and OS outcomes compared to previous research. This favorable outcome can be attributed to a higher proportion of patients (enrolled between 2005 and 2018) receiving advanced systematic treatments, indicating that the incidence of postmastectomy recurrence in pT3N1M0 breast cancer patients is not inherently high due to advancements in systemic therapy.

Furthermore, we observed a strong association between advanced age (>60 years) and decreased OS. This finding is consistent with previous research by McCammon et al.,^21^ Francis et al.,^22^ and Almahariq et al.,^19^ consistently demonstrating an association between advanced age and decreased OS among pT3N0M0 breast cancer patients (multivariate analysis of OS: *p* < 0.01, *p* < 0.001, *p* < 0.001, respectively). However, these studies differed in their age stratification. McCammon et al.^21^ and Francis et al.^22^ categorized patients into <50 and ≥50 age groups, while Almahariq et al.^19^ classified patients into <45, 45–65, and >65 age groups. Furthermore, subgroup analyses conducted by McCammon et al.^21^ showed a substantial improvement in 10‐year OS among women who underwent PMRT, particularly those aged >50 years (70.7% vs. 58.4%, *p* < 0.001). Despite ongoing efforts,^23^, ^24^ a consensus on specific age groups benefiting substantially from PMRT remains elusive. Further investigations are warranted to address this crucial knowledge gap.

To mitigate the risk of undue extrapolation of outcomes in a retrospective analysis within a heterogeneous cohort, we conducted subgroup analysis to explore potential variations in the effects of PMRT across different subpopulations. PMRT exhibited prognostic differences based on LVI status, showing a pronounced correlation with favorable outcomes in LVI‐positive patients, while its impact remained unclear in LVI‐negative individuals. These findings align with previous research,^5^, ^25^ suggesting that patients with positive LVI status may derive greater benefit from PMRT. Additionally, we observed a marginal improvement in DFS with PMRT in the ER‐negative subgroup, contrasting with a moderate effect in the ER‐positive subgroup. Prior studies have indicated that patients with ER‐negative disease, compared with those with ER‐positive disease, had a worse prognosis.^8^, ^26^, ^27^ These analyses might provide insights into identifying suitable candidates for PMRT in pT3N0‐1M0 breast cancer. For example, NCCN guidelines^28^ recommend PMRT for node‐negative patients with additional risk factors like Grade III histology, ER‐negative, and LVI‐positive. Moreover, the Cambridge Breast Unit (CBU) devised a C‐PMRT index^29^ considering factors like age, histology, LVI, etc., to assist in PMRT decision‐making for pN0‐1 breast cancer patients. However, acknowledging the inherent limitations of our single‐center retrospective analysis with a limited sample size, it is crucial to note that conclusions from studies with constrained samples may not definitively rule out chance occurrences. our study was prone to false positives, undermining result reliability. Furthermore, the exploratory nature of our analysis, with undetermined subgroup quantities and a lack of randomization, introduces uncertainties in our conclusions.

Several studies have questioned the necessity of PMRT in pT3N0‐1M0 breast cancer patients due to their inherently low LRR rates.^17^, ^25^, ^30^ However, our study demonstrates that PMRT significantly improves DFS by reducing mortality and any tumor recurrence, including LRR (Table 4). Specifically, PMRT led to a more than twofold reduction in LRR, particularly in the chest wall and supraclavicular lymph node regions. This beneficial outcome is likely attributed to the majority of patients in the PMRT group receiving radiation targeting these regions. Among the 112 patients who underwent PMRT, all received radiation therapy targeting the chest wall. Of these, 76 patients (67.9%) with identified risk factors of metastasis received additional radiation to the supraclavicular or infraclavicular lymph nodes. Additionally, 30 patients (26.8%) with central/medial tumors and positive axillary lymph nodes received supplementary radiation targeting IMNs. The modest absolute LRR benefit of PMRT may result from an underestimated LRR assessment when distant metastasis is present. Furthermore, pivotal randomized trials have provided compelling evidence that combining whole‐breast irradiation with regional nodal irradiation significantly reduced distant metastasis risk.^31^, ^32^ Thus, relying solely on LRR rate is inadequate for assessing the need for PMRT.

On the other hand, when evaluating the impact of PMRT on pT3N0‐1M0 breast cancer, the potential for radiotherapy‐induced toxicities, such as cutaneous reactions, lymphedema, and potential cardiac or pulmonary complications, must be considered. Technological advancements in radiotherapy have significantly improved efficacy while systematically reducing associated toxicities.^33^, ^34^, ^35^, ^36^, ^37^ In our study, the majority of participants (73.2% of the PMRT group) underwent IMRT, which, compared to three‐dimensional conformal radiotherapy (3DCRT), enhances target dose uniformity and reduces high‐dose exposure to surrounding normal tissues.^36^, ^38^, ^39^ Notably, proton therapy, characterized by heightened precision, holds the potential to further minimize radiation‐induced damage to surrounding normal tissues.^40^, ^41^ Although our investigation specifically utilized photon therapy, exploring the application of postmastectomy proton therapy may yield additional benefits, particularly for patients with left‐sided breast cancer or compromised cardiac function. Moreover, recent research indicates comparable efficacy between hypofractionated and conventional fractionated PMRT, with hypofractionation significantly reducing skin toxicities through a condensed treatment duration.^42^, ^43^ All participants receiving PMRT in our study adhered to a conventional fractionated radiation schedule. The applicability of a hypofractionated PMRT regimen for pT3N0‐1M0 breast cancer warrants further investigation at our center.

Our study possesses several inherent limitations. First, its retrospective nature renders it susceptible to selection biases, which cannot be completely offset by employing multivariate analysis and PSM to adjust potential confounders. Second, the relatively small sample size in our study results in a limited number of observed events, challenging the attainment of robust statistical power and generalizability of our findings. Instances of *p*‐values barely passing the 5% threshold raise concerns about chance‐driven statistical significance, potentially leading to false positives. Additionally, borderline significance levels, commonly observed in small sample sizes, complicate the discernment of true effects or associations. While our findings share some similarities with comparable studies, caution is warranted, and future research with larger sample sizes is imperative for validation. Lastly, it is crucial to acknowledge the heterogeneity of treatments received by the patients, including variations in chemotherapeutic regimens and PMRT target volumes. These differences in treatment approaches may have influenced treatment outcomes and introduced additional complexity in interpreting the results.

In conclusion, our investigation reveals favorable overall prognoses among patients with pT3N0‐1M0 breast cancer treated with state‐of‐the‐art therapy. Despite the seemingly modest absolute gains, PMRT leads to a reduction in LRR by more than twofold, significantly enhancing both DFS and OS for these individuals. However, the impact of PMRT appears to vary among patients with different risk statuses. Given the retrospective design of our study and the relatively limited sample size, further investigation is imperative to validate and enhance these initial findings.

## AUTHOR CONTRIBUTIONS

**Xinxin Rao:** Formal analysis (lead); investigation (equal); writing – original draft (lead). **Xuanyi Wang:** Data curation (equal); methodology (equal); software (equal). **Kairui Jin:** Investigation (equal); visualization (equal). **Yilan Yang:** Investigation (equal). **Xu Zhao:** Investigation (equal). **Zhe Pan:** Investigation (equal); software (equal). **Weiluo Lv:** Data curation (equal); investigation (equal). **Zhen Zhang:** Resources (equal); supervision (equal). **Li Zhang:** Data curation (equal); writing – review and editing (equal). **Xiaoli Yu:** Conceptualization (equal); writing – review and editing (equal). **Xiaomao Guo:** Conceptualization (equal); funding acquisition (lead); resources (equal); supervision (lead).

## FUNDING INFORMATION

This work was supported by the Key Clinical Specialty Project of Shanghai.

## ETHICS STATEMENT

The study was conducted with the approval from the Ethics Committee Board of Fudan University Shanghai Cancer Center and written informed consent was waived due to the retrospective nature of the study.

## Supporting information


Figure S1.

## Data Availability

Research data are stored in an institutional repository and will be shared upon request to the corresponding author.
