# Supplementary material for: Outcomes with and without postmastectomy radiotherapy for pT3N0‐1M0 breast cancer: An institutional experience
Source: Cancer Med. 2024 Jan 8;13(1):e6927. doi: 10.1002/cam4.6927 (PMC10807573; doi:10.1002/cam4.6927)
Supplement: Supplementary file 1 — Figure S1. [file CAM4-13-e6927-s001.pdf]

Supplementary Figures

Figure S1

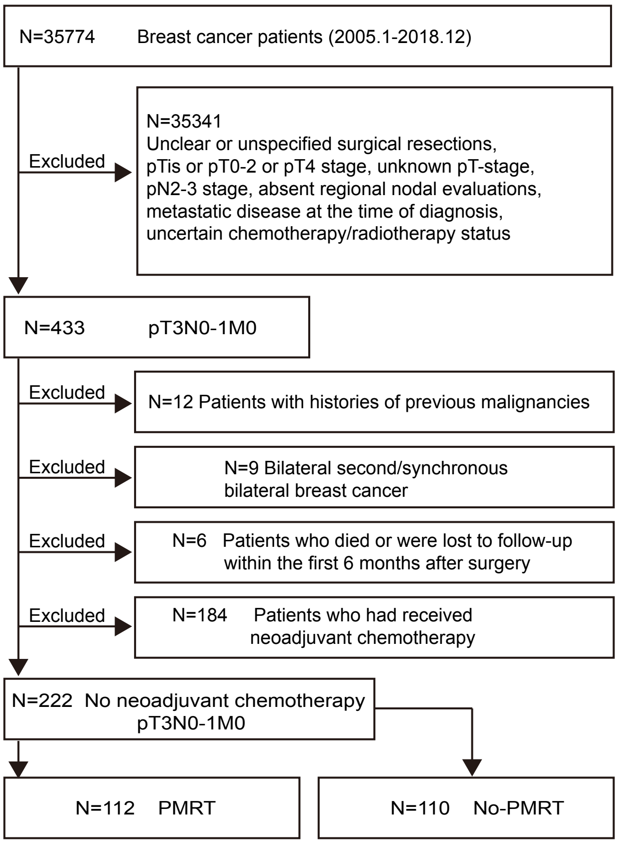

Figure S1. Flow diagram of patient selection for the analysis population.
Abbreviations: PMRT - post-mastectomy radiotherapy.
